# Spinning Cellulose Hollow Fibers Using 1-Ethyl-3-methylimidazolium Acetate–Dimethylsulfoxide Co-Solvent

**DOI:** 10.3390/polym10090972

**Published:** 2018-09-01

**Authors:** Linfeng Lei, Arne Lindbråthen, Marius Sandru, Maria Teresa Guzman Gutierrez, Xiangping Zhang, Magne Hillestad, Xuezhong He

**Affiliations:** 1 Department of Chemical Engineering, Norwegian University of Science and Technology, NO-7491 Trondheim, Norway; linfeng.lei@ntnu.no (L.L.); arne.lindbrathen@ntnu.no (A.L.); maria.t.g.gutierrez@ntnu.no (M.T.G.G.); magne.hillestad@ntnu.no (M.H.); 2 SINTEF Industry, SINTEF AS, NO-7465 Trondheim, Norway; marius.sandru@sintef.no; 3 Beijing Key Laboratory of Ionic Liquids Clean Process, Institute of Process Engineering, Chinese Academy of Sciences, P.O. Box 353, Beijing 100190, China; xpzhang@ipe.ac.cn

**Keywords:** ionic liquids, cellulose, hollow fiber, spinning, viscosity

## Abstract

The mixture of the ionic liquid 1-ethyl-3-methylimidazolium acetate (EmimAc) and dimethylsulfoxide (DMSO) was employed to dissolve microcrystalline cellulose (MCC). A 10 wt % cellulose dope solution was prepared for spinning cellulose hollow fibers (CHFs) under a mild temperature of 50 °C by a dry–wet spinning method. The defect-free CHFs were obtained with an average diameter and thickness of 270 and 38 µm, respectively. Both the XRD and FTIR characterization confirmed that a crystalline structure transition from cellulose I (MCC) to cellulose II (regenerated CHFs) occurred during the cellulose dissolution in ionic liquids and spinning processes. The thermogravimetric analysis (TGA) indicated that regenerated CHFs presented a similar pyrolysis behavior with deacetylated cellulose acetate during pyrolysis process. This study provided a suitable way to directly fabricate hollow fiber carbon membranes using cellulose hollow fiber precursors spun from cellulose/(EmimAc + DMSO)/H_2_O ternary system.

## 1. Introduction

Membrane systems possess many advantages, such as small footprint, low capital and operating costs, being environmentally friendly, no-moving part for the separation as such, and exhibiting process flexibility, which attracts great interest in different gas separations, such as air separation, natural gas sweetening, and biogas upgrading. Most of commercial membranes for gas separation today are polymeric membranes. However, polymer membranes have several limitations for the application in harsh conditions, such as natural gas sweetening, and their relatively low separation performance was found to be caused by membrane compaction and plasticization. Carbon membranes with high mechanical strength can potentially operate at high pressure without having significant loss of separation performance. Carbon membranes are ultra-microporous inorganic membranes prepared mainly by carbonization of polymeric precursors, and typically form a graphitic or turbostratic structure, which presents high mechanical strength and moderate modulus compared to the graphitized fibers. Different polymeric precursors, mainly polyimides (PI) and cellulose derivatives, have been used for preparation of carbon membranes [1,2,3]. Our previous cellulosic-derived carbon membranes showed high CO_2_/CH_4_ selectivity, but relatively low CO_2_ permeance (<0.1 m^3^(STP)/(m^2^·h·bar)) [4]. Moreover, the cellulosic-derived carbon membranes have major fabrication challenges related to fiber drying after deacetylation of the cellulose acetate (CA) hollow fiber precursors [5]. Thus, preparation of carbon membranes directly from cellulose hollow fibers (CHFs) could be a potential solution to address the challenges for high performance carbon membrane development. 

Processing cellulose is, however, difficult in general, because this natural polymer material is not soluble in conventional solvents (e.g., *N*-methyl pyrrolidone, NMP) due to its inter- and intra-hydrogen bonding, and semicrystalline structure. Ionic liquids (ILs), as a new type of green solvent, provide the possibility to dissolve cellulose [6,7] and lignocellulosic biomass [8,9] in a clean process, which has attracted a great deal of academic and industrial interest. Compared to conventional solvents, ILs show remarkable properties, such as a very low vapor pressure, high thermal stability, non-volatility, and low eco-toxicity. Moreover, by proper selection of cation–anion combinations, ILs can be designed with desired properties (e.g., viscosity, cellulose solubility, and eco-toxicity) [10,11]. Among them, 1-butyl-3-methylimidazolium chloride ([Bmim]Cl)) has been reported to dissolve lignocellulosic biomass at above glass transition temperature (180 °C) and spin cellulose fibers [12,13]. However, spinning hollow fibers under such high temperatures is not suitable, due to the difficulty of controlling the temperature of the dope solution reservoir and coagulation bath. In order to reduce dope solution viscosity and, thus, spin hollow fibers at a mild temperature, the solvent dimethylsulfoxide (DMSO) was introduced as co-solvent to decrease the solution viscosity. It was suggested that DMSO, as an aprotic solvent, could partially break down the ionic interaction of [C_4_mim]^+^ and [CH_3_COO]^−^ by solvation of the cation and anion [14], resulting in an accelerated dissolution process and decreased viscosity. Moreover, adding DMSO into the solvent for cellulose dissolution is an economical way to reduce hollow fiber production cost. A mixed solvent of ionic liquid [Bmim]Cl and DMSO was reported to spin cellulose hollow fibers (CHFs) by dry–wet spinning method at 80 °C, using a dope solution of 9 wt % cellulose in [Bmim]Cl-DMSO (weight ratio was 3:1) [15]. The prepared hollow fiber membranes showed a potential application in wastewater treatment. Recently, cellulose flat sheet membranes were fabricated by phase inversion using 1-ethyl-3-methylimidazolium acetate (EmimAc) and EmimAc-DMSO [16]. By coagulation in different non-solvents (water and ethanol), the membranes showed different surface morphologies and blue dextran rejections. However, to date, there are no studies that have reported CHF spinning under moderate/mild temperature (e.g., <50 °C) based on a green spinning process. Thus, exploring the dissolution of cellulose at lower temperature and spinning CHFs are essential to simplify hollow fiber carbon membrane preparation procedure and reduce production cost for future upscaling and commercialization.

In the present work, the EmimAc-DMSO co-solvent was chosen to dissolve microcrystalline cellulose (MCC), and then spin hollow fibers at a mild temperature by dry–wet spinning method. In order to evaluate the suitability of their applications in making hollow fiber carbon membranes, the morphology, structure, and thermal properties of regenerated CHFs were comparatively characterized by different methods. This work presents a unique green approach for the fabrication of CHFs.

## 2. Materials and Methods

### 2.1. Materials

MCC (Avicel PH-101, particle size ~50 µm) and DMSO (Feed Grade, ≥99%) were purchased from Sigma-Aldrich, Oslo, Norway, and used as received. The ionic liquid of EmimAc was provided by the Institute of Process Engineering, Chinese Academy of Sciences (IPE-CAS). Prior to the use, EmimAc was washed by ethyl acetate to remove the impurities, and the pure IL structure was confirmed by ^1^H and ^13^C NMR [17]. Tap water was used as non-solvent in the coagulation and rinsing baths. Ethanol (96% pure) was bought from Sigma-Aldrich, Oslo, Norway, for final solvent exchange, before drying of hollow fibers.

### 2.2. Dope Solution Preparation

The MCC and EmimAc were dried at 80 °C under vacuum (ca. 850 mbar) for 72 h to remove adsorbed water before making dope solutions. The co-solvent of 75 wt % EmimAc/25 wt % DMSO was used to dissolve cellulose. A given amount of MCC was gradually added into the co-solvent under mechanical stirring (500 rpm) at 50 °C for 12 h. The dope solution of 10 wt % cellulose in (EmimAc + DMSO) was prepared inside a nitrogen glove box to avoid water adsorption. 

### 2.3. Spinning CHFs

**Figure 1:**
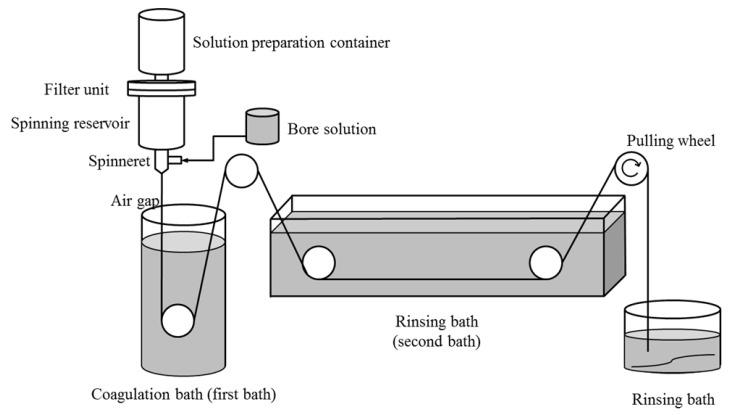
Schematic diagram of the spinning process.

A typically dry–wet method spinning process is illustrated in Figure 1. The pre-conditioning of dope solution was conducted by removing any solid impurities through a porous stainless steel filter. To ensure an air bubble-free dope solution, the filtered dope solution was degassed in the spinning reservoir for 24 h. It was worth noting that filtration and degassing steps are crucial for hollow fiber spinning, as undissolved polymers or air bubbles present in the dope solution will lead to the formation of defects or macrovoids inside the hollow fibers. The extrusion rates of dope solution and bore solution (20 wt % water/60 wt % EmimAc/20 wt % DMSO) were controlled by two gear pumps at different ratios. A double spinneret with outer diameter of 0.7 mm and inner diameter of 0.5 mm was used for spinning hollow fibers. A rubber pulling wheel was employed to control the take-up speed of spun fibers. The spinning parameters have significant influences on the hollow fiber morphology, structure, and properties [18], and should be optimized to obtain suitable hollow fibers for making carbon membranes. The spinning condition listed in Table 1 was used to fabricate defect-free CHFs. Following the hollow fiber spinning, the obtained hollow fibers were placed in a water bath overnight to remove excess solvent. The hollow fibers were finally dried at room temperature and in a dust free area for subsequent characterization and carbon membrane fabrication. 

### 2.4. Characterization

Water adsorption behavior of EmimAc was investigated by Fourier transform infrared (FTIR) spectra analysis (Nicolet iS50 FTIR Spectrometer, Thermo Scientific, Oslo, Norway) and thermogravimetric analysis (TGA) using TG 209F1 Libra from Netzsch, Malmø, Sweden. The rheological properties of cellulose/IL solutions were measured by a Discovery HR-2 Rheometer from TA Instruments, Oslo, Norway, equipped with a 20 mm parallel plate geometry and Peltier Plate Steel temperature control system. A low viscosity silicon oil (~20 mPa·s) was used to minimize the effort of water absorption into the solutions during analysis. An Axio Imager A1 optical microscope from Carl Zeiss, Oslo, Norway, was used to investigate the dissolving process with polarized and non-polarized light, and preliminarily analyze the morphology of wet hollow fibers. The structure and morphology of dried hollow fibers were characterized by scanning electron microscope (SEM) from Hitachi High Technology, Lidingø, Sweden (TM3030 tabletop microscope). The SEM samples were prepared in liquid nitrogen, and then sputter-coated with gold layer. 

FTIR analysis of the solutions and celluloses were preformed to characterize the chemical structures with a wave number range of 650 to 4000 cm^−1^ under ambience. The thermal stabilities of MCC and CHF were investigated by TGA with a heating rate of 2 °C/min from room temperature to 600 °C under nitrogen flow. The degree of crystallinity of the MCC and regenerated CHF samples were determined by X-ray diffraction (XRD) method. The scans were conducted from 5° to 60° on Bruker D8 A25 DaVinci X-ray Diffractometer, Solna, Sweden with Cu Kα radiation. The crystallinity index (CrI) of celluloses were estimated by the following empirical equation [19]:

(1) $$\mathrm{Crl}=\frac{I_{total,}-I_{am}}{I_{total}}\times 100\%$$

where $I_{total}$ is the intensity at the main peak and $I_{am}$ is the minimum intensity between the main and secondary peaks.

## 3. Results and Discussion

### 3.1. Water Adsorption in EmimAc

**Figure 2:**
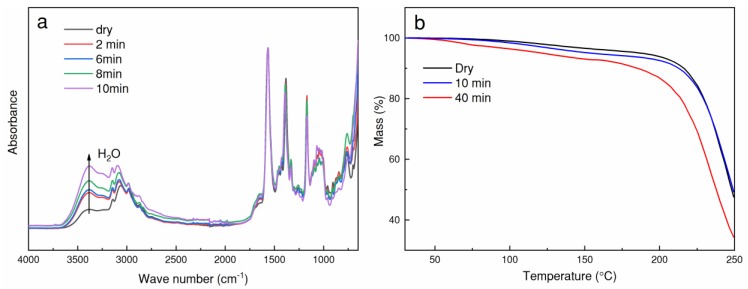
FTIR spectra (**a**) and TGA curves (**b**) of 10 wt % cellulose/EmimAc solution exposed to ambient air.

In order to document water adsorption behavior, a 10 wt % MCC/EmimAc solution was prepared for the FTIR and TGA analysis. The sample solution was exposed to ambient air for different time, and the results are shown in Figure 2. It can be seen that the amount of adsorbed water in the solution significantly increases over time, based on the increased intensity of the –OH group at the wave number of 3400 cm^−1^ (i.e., the characteristic peak of H_2_O). Moreover, higher weight loss of the solution exposed for longer time (e.g., 40 min) was found compared to that with the short time exposure. Both FTIR and TGA results proved a strong water adsorption behavior in EmimAc. A rheological study regarding water content in native cellulose/IL solutions revealed tiny amounts of water (~0.25 wt %) could have a significant effect on the rheological properties [20]. Moreover, the presence of water in the solvent can affect the dissolution behavior. Liu et al., reported that the ratio of IL and water can improve the gelatinization or dissolution of corn starch in [MMIM][(MeO)·HPO_2_] [21]. In this work, to avoid the risk of viscosity increase and precipitation of cellulose in the spinneret, preparation of cellulose/EmimAc solution was conducted in a nitrogen glove box, to precisely control the solution composition for the subsequent spinning. 

### 3.2. Viscosity of Cellulose/IL Solution

**Figure 3:**
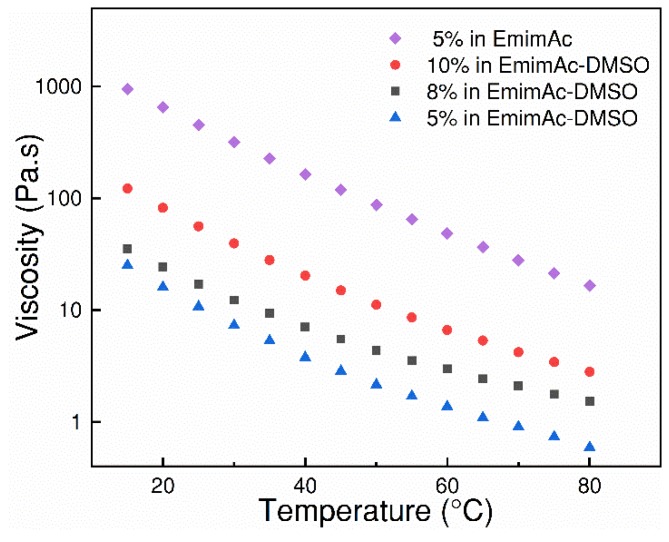
The viscosities of different cellulose/ionic liquid (IL) solutions as a function of temperature.

The viscosity of dope solution is crucial for hollow fiber spinning, and should be lower than 270 Pa∙s, according to our practical experience, for the actual spinning equipment used. If the viscosity of dope solution is higher than 270 Pa∙s, it can be very difficult to extrude the dope solution continuously. This is because the high viscosity will lead to inlet starvation of the pump, and will thus cause the trapping of air bubbles in the inlet of spinneret. For a different spinning rig, the value might be different. Figure 3 shows the viscosity of cellulose/EmimAc and cellulose/(EmimAc + DMSO) solutions with different composition. It can be seen that the viscosities of different cellulose/(EmimAc + DMSO) solutions decreases with the increase of temperature. A much lower viscosity for cellulose/(EmimAc + DMSO) system was obtained compared to the corresponding cellulose/EmimAc solution without DMSO. It was also found that the 10 wt % cellulose/(EmimAc + DMSO) solutions has an obviously lower viscosity of ~12 Pa·s than 5 wt % cellulose/EmimAc solution of ~115 Pa·s at 50 °C. The reason is that DMSO can partially break down the ionic association between [C_4_mim]^+^ and [CH_3_COO]^−^ to produce more free [CH_3_COO]^−^ anions, and thus increase the dissolution rate and reduce viscosity, as reported by Zhao et al. [14]. For the dry–wet spinning process, a dope solution with suitable viscosity and high polymer concentration is usually required, as the low viscosity guarantees the process feasibility, and high polymer concentration ensures the high mechanical strength of hollow fibers [15]. Thus, a 10 wt % MCC/(EmimAc + DMSO) dope solution was chosen for hollow fiber spinning at 50 °C. 

### 3.3. Morphology of CHFs 

**Figure 4:**
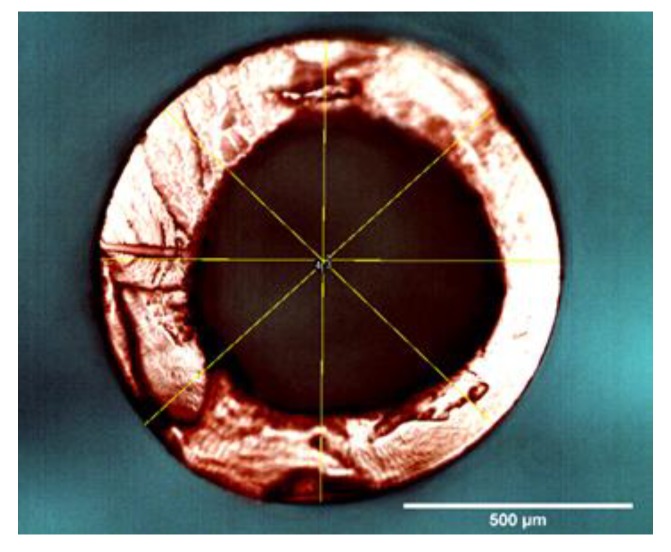
Cross-sectional image of a wet CHF obtained from optical microscope.

**Figure 5:**
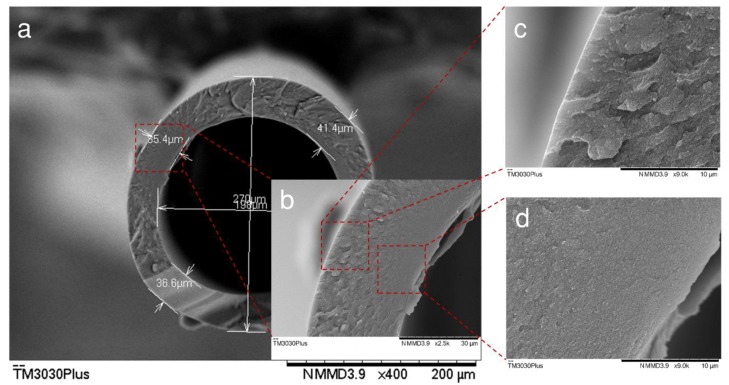
SEM images of (**a,b**) cross section of CHF; (**c**) outside layer of CHF; (**d**) inside layer of CHF.

Various spinning conditions were employed to fabricate different cellulose hollow fibers (see the Supplementary Materials), and the CHFs spun under the conditions listed in Table 1 were identified as the best precursors for further characterization in this work. During the spinning process, the morphology of wet hollow fibers was preliminary investigated by the optical microscope. A typical cross-sectional image for a wet CHF is displayed in Figure 4. The average diameter and thickness of wet CHFs were found to be ca. 1000 and 160 µm, respectively. The morphology of dried CHFs was characterized by SEM, and Figure 5 shows the cross-sectional images of the dried CHFs. The hollow fibers showed significant shrinkages after drying with an average outer diameter and thickness of 270 and 38 µm, respectively (Figure 5a). It can be seen that the spun CHFs formed a defect-free structure. During the spinning process, a delayed demixing occurred as the starting point of dope solution composition was far away from the binodal curve. Therefore, the regenerated cellulose chains move close to each other, and result in a dense structure as shown in Figure 5b. It was also found that the outer and inner structure of hollow fibers were only a little different, as shown in Figure 5c,d, which is attributed to the (EmimAc + DMSO)/H_2_O mixture used in the bore solution, instead of water in the coagulation bath. However, no significant difference in porosity can be seen from the SEM images. It should be noted that the SEM images reported in this work have relatively low resolution, and thus it is impossible to see the different structure on that scale. The dense structure of the spun CHFs shows a potential to be used as suitable precursors for making carbon membranes subsequently, or membranes for specific separation processes. 

### 3.4. FTIR Analysis of CHFs 

**Figure 6:**
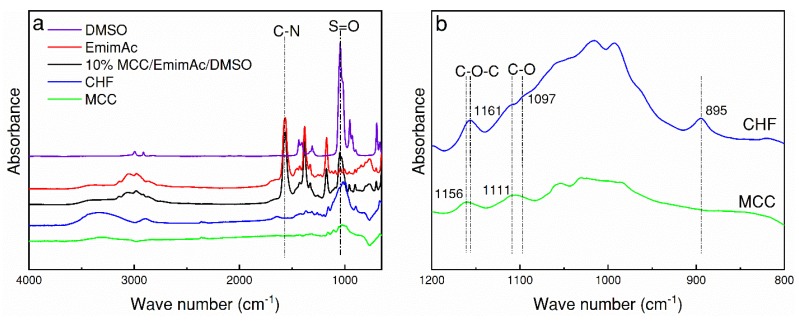
FTIR spectra of (**a**) solvents, polymer solution, MCC and CHF; (**b**) zoom in the range of 800-1200 cm^-1^ for MCC and CHF.

Figure 6 presents the FTIR spectra of solvents, polymer solutions, MCC, and CHFs. Compared to the 10 wt % dope solution, the characteristic peaks of CHFs at position 1570 and 1039 cm^−1^, corresponding to the C=N bond in EmimAc and S=O bond in DMSO, disappear. This implies that most of the co-solvent was removed during the coagulation and solvent-exchanging process. Moreover, a bond shifting was observed between MCC and CHFs, as shown in Figure 6b, with a narrow wave number in the range of 800–1200 cm^−1^. The peak of MCC at 1161 cm^−1^, corresponding to C–O–C stretching vibrations, shifts to 1156 cm^−1^ in CHFs, which indicates that the cellulose I crystalline structure in MCC was transformed to cellulose II in the CHFs during the spinning process, as has been reported in the literature [22]. The stretching of C–O bond located at 1111 cm^−1^ in MCC shifts to 1097 cm^−1^ in the regenerated CHF. Moreover, the characteristic peak of the C–O–C stretching at the β-(1 ⟶ 4)-glycosidic linkage appears at 895 cm^−1^ in CHFs) also proved the cellulose crystalline structure transition [22,23,24]. 

### 3.5. Crystallinity of CHFs

**Figure 7:**
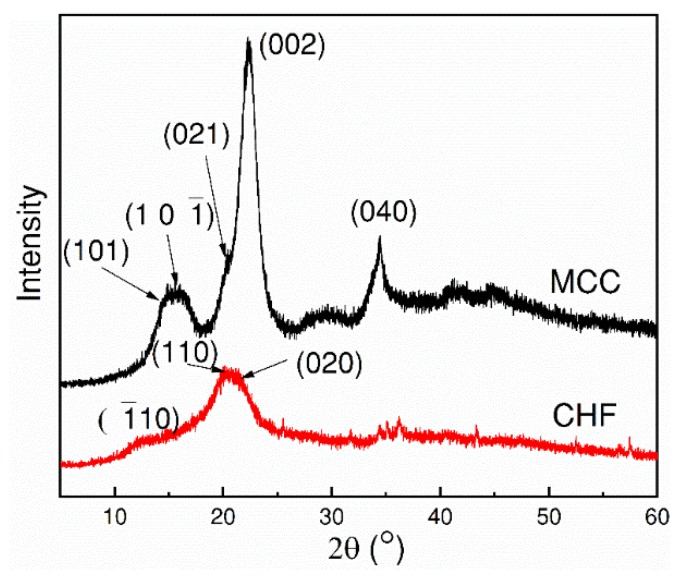
The XRD patterns of MCC and CHF.

In order to document the crystalline structure transition reported in the literature [25], XRD analysis on MCC and the regenerated CHFs was conducted, and the results are shown in Figure 7. The XRD patterns illustrated the diffraction patterns of the samples, whereas the corresponding crystallinity can be estimated by the Equation (1). The main characteristic peaks of MCC in XRD patterns are 2θ = 14.7°, 16.5°, and 22.6°, which correspond to the reflection planes (1 0 1), (1 0 1), and (0 0 2), respectively [26,27,28]. However, the spun CHFs showed a different XRD pattern. After spinning, the main peaks at 14.7°, 16.2°, and 22.6° of MCC disappear, but new peaks appear at 12.2°, and a doublet at 20.2° and 21.5°, which correspond to the reflection planes (1 1 0), (1 1 0), and (0 2 0). This result indicates that the cellulose structure was transited to cellulose II, which was probably caused by the easy association or aggregation of cellulose chains in ionic liquid solution, or during the spinning process, as reported in the literature [26,28,29,30,31]. In this case, a suggestion for the mechanism of crystalline structure transition is described as follows: the MCC (cellulose I) was dissolved in EmimAc/DMSO co-solvent, by disrupting the intermolecular hydrogen bonds between cellulose molecules. Then, the IL molecules were intercalated into the gap between the hydrogen-bonded molecular sheets, resulting in a disordered cellulose [25,30,32]. In the spinning (cellulose regeneration) process, the IL was exchanged with a non-solvent (water), and new hydrogen bonds between cellulose sheets were formed in cellulose Ⅱ. It should be noted that the XRD data obtained in this work were slightly different from that reported in the literature, as both the particle size and the water content can affect the peak positions. 

For cellulose I of MCC, the main and secondary peaks are (0 0 2) and (1 0 1), located at 22.6° and 16.2°, respectively, while for cellulose II, the main peak is a doublet of (1 1 0) and (0 0 2) planes, corresponding to 2θ = 20.2° and 21.5°. The secondary peak appears at (1 1 0) plane at 2θ = 12.2°. The crystallinity index of MCC and CHFs were calculated as 82.0% and 66.7%, respectively. It was worth noting that the degree of crystallinity of the CHFs decreases compared to the raw MCC, which was probably due to the fact that the original crystalline structure and the glycosidic linkages between the glucose units had been partially destroyed or modified during the dissolving and spinning processes by interaction with ionic liquids, which resulted in the reduction of the crystallinity, as reported in the literature [25,33].

### 3.6. Thermal Analysis of CHFs 

**Figure 8:**
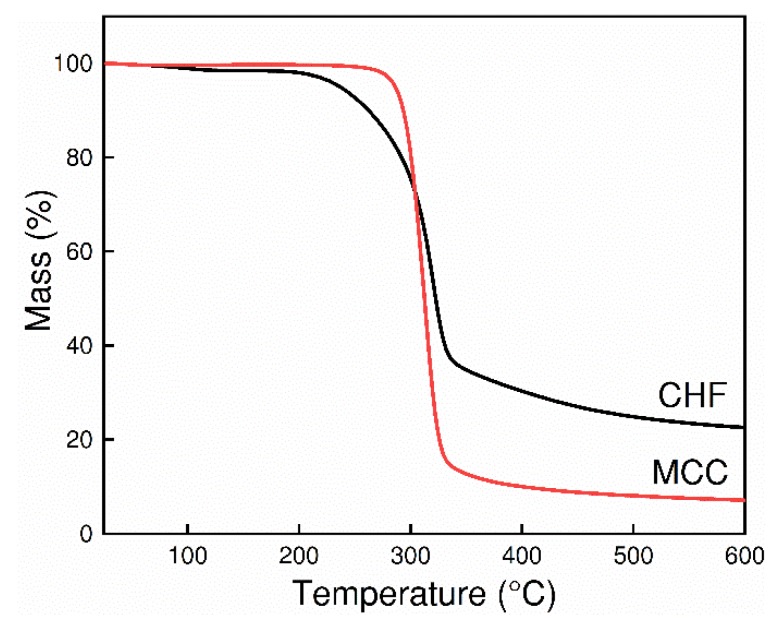
Weight loss of MCC and CHF by TGA.

The TGA curves of MCC and CHFs are shown in Figure 8. A steep decomposition of MCC was found in a narrow temperature range from 280 to 330 °C, and only 7.3% carbon residues was obtained after the pyrolysis. However, the weight loss curve of CHFs was slightly different from that of MCC, and both the processes of water desorption and polymer degradation were observed in CHFs. At low temperatures of 50–200 °C, a small weight loss was found due to the water desorption, and caused by the hydrophilic behavior of CHF polymers [33]. It should be noticed that CHFs presented a lower decomposition temperature and a wider decomposition temperature range, due to the decreased crystallinity of cellulose II. Moreover, due to the transition of crystalline structure, the CHF retained a higher carbon yield of 22.9%. This shows a similar pyrolysis behavior with the deacetylated cellulose acetate precursors reported in the previous work [4,34]. During pyrolysis of the TGA measurement, the water molecules generated from cellulose dehydration reaction and other volatiles, like CO, CO_2_, and CH_4_, eliminated from the ring opening of glycoside molecules, could accelerate the conversion of the cellulosic structure to a turbostratic carbon structure. Thus, the produced CHFs can be used as suitable precursors for preparation of hollow fiber carbon membranes by employment of a suitable carbonization procedure. 

## 4. Conclusions

In this work, the ionic liquid of EmimAc was identified as a good solvent for cellulose dissolution, and defect-free CHFs were successfully fabricated from a dope solution of 10 wt % cellulose in EmimAc-DMSO under 50 °C. A bore flow rate of 1.7 mL/min, a dope flow rate of 3.2 mL/min, a take-up speed of 14.6 m/min, an air gap of 50 mm, and coagulation and rinsing bath temperature of 25 ± 1 °C were found to be suitable for spinning dense CHFs with a typical fiber diameter and thickness of 270 and 38 µm. Moreover, DMSO was confirmed to be a good co-solvent for cellulose dissolution, which reduces the viscosity of dope solution to increase the spinning processability. The crystalline structure transition from cellulose I to cellulose II occurred during the dissolution and spinning process, as shown from the FTIR spectra and XRD patterns. The regenerated CHFs presented similar pyrolysis properties with deacetylated cellulose acetate from thermal analysis, which indicates that cellulose-based hollow fiber carbon membranes can be directly prepared from the CHF precursors. Future work on the optimization of CHFs with desired structure and properties, by controlling spinning condition and dope solution composition, should be conducted to develop high performance carbon membranes for gas separation. 

## Figures and Tables

**Table 1:** List of spinning condition.

Spinning Parameter	Value
Dope flow rate, mL/min	3.2
Bore fluid flow rate, mL/min	1.7
Bore fluid composition, wt %	60 EmimAc + 20 DMSO + 20 H_2_O
Air gap, mm	50
Take-up speed, m/min	14.6
Coagulation and rinsing bath temperature, °C	25 ± 1

## Supplementary Materials

The following are available online at http://www.mdpi.com/2073-4360/10/9/972/s1, Figure S1: SEM images of spun cellulose hollow fibers, Table S1: Different spinning conditions.
