# Supplementary material for: Spinning Cellulose Hollow Fibers Using 1-Ethyl-3-methylimidazolium Acetate–Dimethylsulfoxide Co-Solvent
Source: Polymers (Basel). 2018 Sep 1;10(9):972. doi: 10.3390/polym10090972 (PMC6404078; doi:10.3390/polym10090972)
Supplement: Supplementary file 1 [file polymers-10-00972-s001.pdf]

Supporting Information for

Spinning Cellulose Hollow Fibers Using 1-Ethyl-3-Methylimidazolium Acetate - Dimethylsulfoxide Co-solvent

Linfeng Lei[1], Arne Lindbråthen[1], Marius Sandru[2], Maria Teresa Guzman Gutierrez[1], Xiangping Zhang[3], Magne Hillestad[1], Xuezhong He[1,*]

[1] Department of Chemical Engineering, Norwegian University of Science and Technology, NO-7491, Trondheim, Norway; lingfen.lei@ntnu.no (L.L.); arne.lindbrathen@ntnu.no (A.L.); maria.t.g.gutierrez@ntnu.no (M.T.G.G.); magne.hillestad@ntnu.no (M.H.)

[2] Department of Sustainable Energy Technology, Materials and Chemistry, SINTEF, 7491 Trondheim, Norway; marius.sandru@sintef.no (M.S.)

[3] Beijing Key Laboratory of Ionic Liquids Clean Process, Institute of Process Engineering, Chinese Academy of Sciences, P.O. Box 353 Beijing, 100190, China; xpzhang@ipe.ac.cn (X.Z.)

[*] Corresponding author: xuezhong.he@ntnu.no (X.H.), Tel.: +47 73593942

Table. S1 Different spinning conditions

Run No.	Flow rate (ml/min)		Solvent in bore solution (%)	Wheel speed (m/min)	Air gap (mm)	Coagulation Temperature (°C)	
	Dope	Bore				First bath	Second bath
1	3.2	2.0	90%	14.9	25	46.8	45.5

2	1.6	1.0	90%	14.9	25	46.8	45.5
3	1.6	1.0	90%	17.8	50	47.4	42.0
4	1.6	1.0	80%	14.6	50	26.0	24.0
5	3.2	1.7	80%	14.6	50	26.0	24.0
6	4.8	2.6	80%	17.8	50	26.0	24.0

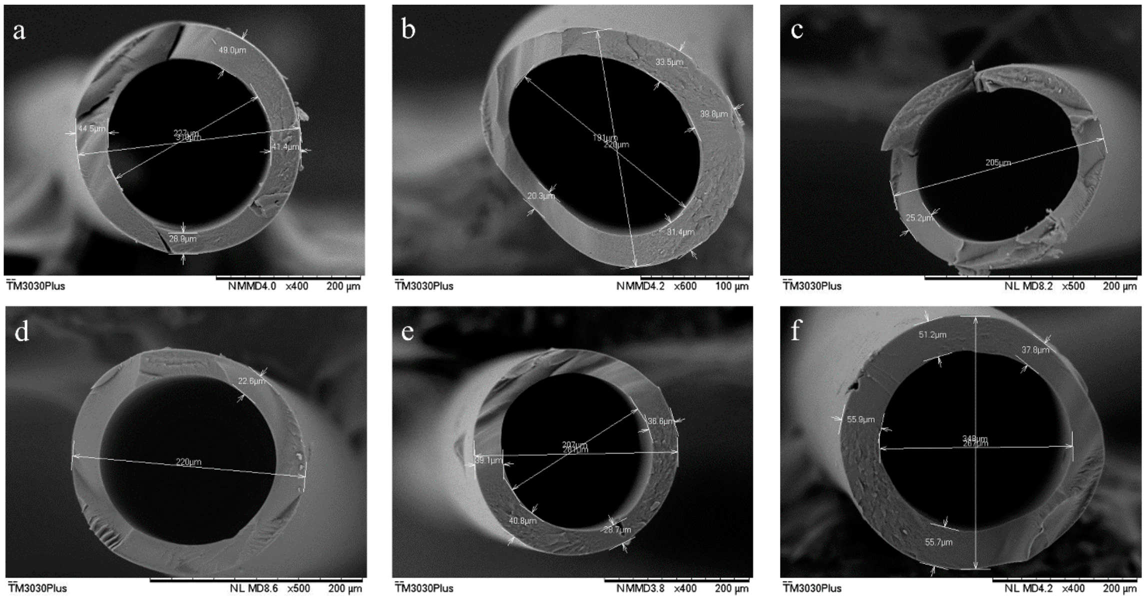

Fig. S1 SEM images of spun cellulose hollow fibers
